# Assessment of proximal pulmonary arterial stiffness using magnetic resonance imaging: effects of technique, age and exercise

**DOI:** 10.1136/bmjresp-2016-000149

**Published:** 2016-10-07

**Authors:** Jonathan R Weir-McCall, Anu Kamalasanan, Deidre B Cassidy, Allan D Struthers, Brian J Lipworth, J Graeme Houston

**Affiliations:** 1 Division of Cardiovascular and Diabetes Medicine, Medical Research Institute, University of Dundee, Dundee, UK; 2 Department of Clinical Radiology, Ninewells Hospital and Medical School, Dundee, UK; 3 Scottish Centre for Respiratory Research, Medical Research Institute, University of Dundee, Dundee, UK

**Keywords:** Imaging/CT MRI etc

## Abstract

**Introduction:**

To compare the reproducibility of pulmonary pulse wave velocity (PWV) techniques, and the effects of age and exercise on these.

**Methods:**

10 young healthy volunteers (YHV) and 20 older healthy volunteers (OHV) with no cardiac or lung condition were recruited. High temporal resolution phase contrast sequences were performed through the main pulmonary arteries (MPAs), right pulmonary arteries (RPAs) and left pulmonary arteries (LPAs), while high spatial resolution sequences were obtained through the MPA. YHV underwent 2 MRIs 6 months apart with the sequences repeated during exercise. OHV underwent an MRI scan with on-table repetition. PWV was calculated using the transit time (TT) and flow area techniques (QA). 3 methods for calculating QA PWV were compared.

**Results:**

PWV did not differ between the two age groups (YHV 2.4±0.3/ms, OHV 2.9±0.2/ms, p=0.1). Using a high temporal resolution sequence through the RPA using the QA accounting for wave reflections yielded consistently better within-scan, interscan, intraobserver and interobserver reproducibility. Exercise did not result in a change in either TT PWV (mean (95% CI) of the differences: −0.42 (−1.2 to 0.4), p=0.24) or QA PWV (mean (95% CI) of the differences: 0.10 (−0.5 to 0.9), p=0.49) despite a significant rise in heart rate (65±2 to 87±3, p<0.0001), blood pressure (113/68 to 130/84, p<0.0001) and cardiac output (5.4±0.4 to 6.7±0.6 L/min, p=0.004).

**Conclusions:**

QA PWV performed through the RPA using a high temporal resolution sequence accounting for wave reflections yields the most reproducible measurements of pulmonary PWV.

**Key messages**

- PWV calculation using MRI is a promising technique for the non-invasive measurement of arterial stiffness within the pulmonary circulation.
- Pulmonary pulse wave velocity is measured most reproducibly in the right pulmonary artery using a single slice flow area technique.
- These measurements are robust to changes in cardiac output states.

## Background

The compliance of the pulmonary artery (PA) is a key component in decoupling the right ventricle from the pulmonary bed, allowing the right ventricle to work at maximum efficiency and protecting the microcirculation from large pressure gradients.^1–3^ Indeed the stiffness of the PA is a strong determinant of right ventricular (RV) function,^4^ and increased stiffness causes distal pulmonary arterial endothelial dysfunction and inflammation.^5^ ^6^ Increased stiffness is independently associated with reduced functional capacity^7^ and higher mortality than the PA pressures or pulmonary vascular resistance.^8–12^ While stiffness is partially dependant on underlying distending pressures,^13^ ^14^ multiple studies have shown the intrinsic stiffness of arteries to be increased in pulmonary hypertension independently of these.^15–19^ Stevens *et al*^15^ demonstrated in patients with pulmonary hypertension a curvilinear relationship between RV function and PA distensibility with extensive loss of PA distensibility before a rapid decompensation of the right ventricle occurred. Pulmonary stiffness is increased early in pulmonary hypertension development and is increased even in those with isolated exercise induced pulmonary hypertension.^17^ ^20–22^ This combination of features suggest pulmonary arterial stiffness as a promising biomarker for detection of early disease and as a potential therapeutic target before end stage arterial remodelling occurs with dire consequences for the failing right ventricle.

The majority of measurements of arterial stiffness require knowledge of the arterial pressures to calculate the stiffness. In the arterial circulation, this is not a significant issue as the brachial arterial pressures are readily obtained with a sphygmomanometer, as mean blood pressure (BP) and diastolic BP are relatively constant throughout the large arteries.^23^ However, in the PAs, this provides a significant hurdle as an external measurement of the pressures is not readily available. Pulse wave velocity (PWV) is an entirely non-invasive technique for measuring arterial stiffness that does not require knowledge of the arterial pressures. This can be measured using MRI and can be assessed using one of two methods: the transit time (TT) technique which measures the time it takes for the pulse wave to travel between two separate points along the vessel; and the flow area (QA) technique which measures the change in cross-sectional area and flow across the vessel at this point to derive the PWV. The results of these techniques have been shown to correlate well with one another in the pulmonary circulation,^24^ and each individual technique has been shown to have good same day interscan reproducibility.^25^ ^26^ However, the reproducibility of the two techniques has not been directly compared in the pulmonary circulation. In addition, the effects of age or physiological flow states on PWV have not been elucidated.

The aim of this study is thus to assess and compare the reproducibility of the two MRI methods for measuring PWV in healthy volunteers, and to assess the effects of age and exercise on these measures.

## Materials and methods

### Population

Two separate study populations were recruited:

1. Young healthy volunteers (YHV): 10 healthy volunteers under 40 (3 males, 7 females, mean age 31.5±2.4 years) with no history of cardiovascular or lung disease were recruited to the study. All individuals underwent high temporal resolution phase contrast scans of their main PA (MPA) and branch PAs at baseline, during exercise and at 6 month follow-up.
2. Older healthy volunteers (OHV): 20 healthy volunteers over the age of 55 (9 male, 11 female, mean age 60.2±1.1 years) with no history of cardiovascular or lung disease were recruited to the study. All individuals underwent high temporal resolution phase contrast scans of their MPA and branch PAs followed by a high spatial resolution phase contrast scan of their MPA. All four sequences were repeated during the same scanning session.

Ethical approval was granted by the East of Scotland Ethics committee 1. All participants gave written informed consent for the study.

### Magnetic resonance imaging

Images were acquired on a 32 RF cardiac receiver channel, 3T MRI Scanner (MAGNETOM Trio, Siemens, Erlangen, Germany). A three-plane localiser was first obtained, following which four-chamber, two-chamber and short-axis localisers of the heart were obtained. An axial half-Fourier acquisition turbo spin echo (HASTE) stack was acquired of the chest. From these a balanced steady state free precession (bSSFP) of the RV outflow tract was planned following which an orthogonal plane was acquired to optimally visualise the MPA and valve. Localisers along the length of the right PA (RPA) and left PA (LPA) were then obtained. From these, phase contrast imaging was acquired in three planes through the MPA, RPA and LPA to provide a true cross section through each of the three arteries. The MPA slice was located as close to the valve as possible in order to maximise the distance for the TT technique while also avoiding the valve throughout the cardiac cycle. The RPA and LPA were placed as close to the hila as possible while remaining proximal to the origins of the first visualised branch.

For the high temporal resolution scan, the acquisition parameters of the phase contrast sequence were as follows: slice thickness=6 mm, repetition time/echo time (TR/TE)=7/4 ms, no. averages=1, phases=128, velocity encoding=150 cm/s, bandwidth/pixel=340 Hz, flip angle=15°, field of view (FOV)=320×320 mm^2^, matrix=256×256. This provided a temporal resolution of 7 ms and a spatial resolution of 1.25×1.25×6 mm. For the high spatial resolution scan, the acquisition parameters were as follows: slice thickness=6 mm, TR/TE=12/4 ms, no. averages=1, phases=80, velocity encoding=150 cm/s, bandwidth/pixel=340 Hz, flip angle=15°, field of view (FOV)=320×320 mm^2^, matrix=512×512. This provided a temporal resolution of 12 ms, spatial resolution of 0.625×0.625×6 mm. Both sequences were free breathing, with an acquisition time of ∼4 min depending on heart rate.

In addition, YHVs and OHVs also underwent aortic PWV assessment of their aortic arch. The high temporal resolution acquisition sequence was performed at the level of the RPA with the distance between the ascending and descending aorta measured on a candy cane view of the thoracic aorta as previously described.^27^

### Exercise

Isometric exercise was performed by the volunteer by crossing their feet and then forcefully plantarflexing the superior foot while dorsiflexing the inferior foot against each other. The participant was instructed that if any discomfort developed, they should swap the positions of the feet and to continue the exercise. Image acquisition began after 5 min of this exercise with the exercise continuing throughout the duration of the image acquisition. Gentle encouragement was given to ensure sustained effort throughout the process.

### Image analysis

The images were exported with image analysis performed using cvi^42^ (Circle Cardiovascular Imaging Calgary, Alberta, Canada).

**Figure 1:**
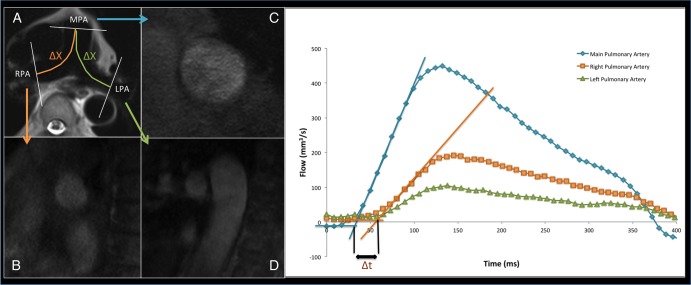
Transit time technique: (A) axial HASTE through the pulmonary trunk bifurcation; (B) short axis RPA; (C) short axis MPA; (D) short axis LPA; (E) flow curves from the three pulmonary arteries. Time delay between arrival of the three flow curves can then be derived, while the distance is measured from the axial HASTE image. HASTE, half-Fourier acquisition turbo spin echo; LPA, left pulmonary artery; MPA, main pulmonary artery; RPA, right pulmonary artery.

For the TT method, distance and time data need to be measured. For the distance, the HASTE axial images were used to measure the distances between the imaging planes following the centreline of the vessel. Where the RPA or LPA lay on different slices from the MPA, a vertical height was calculated from the slice thickness and number of slices. Using this vertical height and horizontal length measured on the axial slices, the final distance was calculated using Pythagoras theorem. For the time component, the phase and magnitude images were pulled up side by side. A contour was manually drawn around the perimeter of the vessel on the magnitude image. This was then automatically propagated throughout the remainder of the images and manually corrected where malposition occurred. The program then automatically calculated area, flow and velocity data which were exported to Excel 2010 (Microsoft, USA). The flow curves from the MPA, RPA and LPA were plotted, and the time to the systolic upstroke of the waves then calculated. The arrival time of the flow wave was identified as the intersection between the systolic upstroke and baseline flow. The systolic upstroke was calculated as the line through the data points that lay between 20% and 80% levels of the maximum flow rate. The baseline was the horizontal line at minimum velocity before systole. Pulse wave was calculated for RPA and LPA using equation 1:1
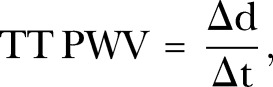


where Δd is the distance between the planes, and Δt is the difference in the time to foot between the MPA and RPA/LPA (see figure 1).

**Figure 2:**
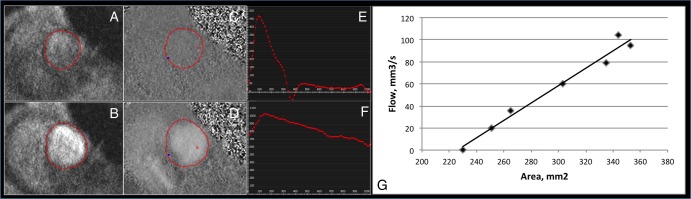
Assessment of the main pulmonary artery for calculation of the QA PWV. (A and C) demonstrate the magnitude and phase contrast images at the start of systole, while (B and D) demonstrate the magnitude and phase images at peak systolic flow. From these, the flow (E) and area (F) of the pulmonary artery is calculated, and then charted against each other (G), with the gradient of this line representing the PWV. QA, flow area; PWV, pulse wave velocity.

For the QA method, the phase and magnitude images were pulled up side by side. A contour was manually drawn around the perimeter of the vessel on the magnitude image on each image for the first 200 ms of the cardiac cycle. From this, the program calculated the total area and flow within the cross section of the pulmonary vessel. The area and flow were then plotted against one another during early systole. Early systole was defined as the time period in systole during which the vessel area and flow were simultaneously increasing. Three techniques have been described for the calculation of QA PWV with all being variations on the basic premise that:
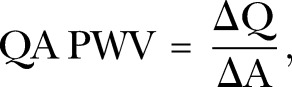


where Q is the flow and A is the area through the PA. The first is as described by Peng *et al*^26^ whereby the gradient of the line is fitted through these points using a minimum squared difference technique hereby known as QA_Trad_ (figure 2). The technique proposed by Quail *et al*^28^ follows the same principle but restricts analysis to the first three data points of the systolic upstroke in order to avoid the influence of reflected waves (QA_3_). Finally, Davies *et al*^29^ have proposed a technique that accounts for effects of reflected waves thereby allowing usage of more data points than the Quail *et al* technique while maintaining accuracy. This was originally described for pressure and velocity data derived from invasive catheter measurements (QA_Inv_), however has been adapted as follows:
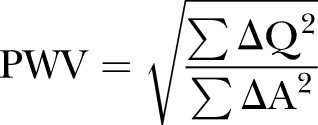


This used all data points in early systole, similar to the QA_Trad_ technique.

### Statistics

Descriptive statistics were used for the analysis of the demographic and clinical features of the cohorts with data expressed as mean±SEM. Normality and equality of variances of the variables were tested. A dependant sample t-test was used to compare the difference between the first scan and the repeated measure, interscan measure and the exercise measure. Analysis of variance (ANOVA) was used to compare PWV between the two cohorts. Bland-Altman plots were used to further investigate the interscan and interobserver reproducibility. All data were analysed using SPSS statistical package (V.21.0, SPSS Chicago, Illinois, USA). Significance was assumed when p<0.05.

## Results

### PWV measurements

Using the TT technique PWV was measurable in all individuals; however, in five individuals, one of the sides produced grossly inaccurate results (PWV excessively high or negative), this error was slightly more common on the left (n=3) than the right (n=2). Using the high temporal resolution QA technique, PWV was measurable in 29/30 of the study participants at the MPA, 29/30 at the RPA and 29/30 at the LPA. Using the high spatial resolution QA technique, PWV was measurable in 29/30 participants.

Using the TT technique, MPA–RPA PWV was 2.7±0.1/ms while the MPA–LPA PWV was 3.3±0.2/ms. There was a significant difference between the PWV using the RPA and the LPA: mean (95% CI) of the differences −0.55 (−1.1 to −0.03), p=0.038.

The results of the three QA techniques for the high temporal resolution and low temporal resolution sequences are described in table 1. Using the high temporal resolution sequences, the QA_Trad_ produced significantly higher results than the QA_3_ (p<0.001) or the QA_Inv_ (p<0.001), while there was no difference between the QA_3_ and QA_Inv_ techniques (p=0.41). Similar findings were observed with the high spatial resolution sequence with the QA_Trad_ produced significantly higher results than the QA_3_ (p=0.004) or the QA_Inv_ (p<0.001), while there was no significant difference between the QA_3_ and QA_Inv_ techniques (p=0.47).There was no difference in PWV between the two sequences using QA_Trad_; however, the higher temporal resolution yielded consistently lower PWV than the high spatial resolution sequence for the QA_3_ (p=0.028) and QA_Inv_ (p=0.001). Using the QA_Trad_ method, the PWV was 1.99±0.14/ms in the MPA, 1.49±0.11/ms in the RPA and 1.37±0.11/ms in the LPA. The PWV was significantly higher in the MPA compared the RPA (mean (95% CI) of the differences: 0.49 (0.15 to 0.89), p=0.006) and the LPA (mean (95% CI) of the differences 0.63 (0.36 to 0.89), p<0.001). There was no significant difference between the RPA and LPA (mean (95% CI) of the differences 0.12 (−0.11 to 0.34), p=0.29).

**Table 1:** Comparison of PWV between the two phase contrast sequences and three postprocessing techniques

	QA_Trad_	QA_3_	QA_Inv_
High temporal resolution	1.98±0.13	1.0±0.11	1.1±0.08
High spatial resolution	2.11±0.17	1.55±0.16	1.67±0.13

3, three point technique; Inv, inverse squared reciprocal technique; PWV, pulse wave velocity; QA, flow area PWV; Trad, traditional technique.

### Within-scan reproducibility

**Figure 3:**
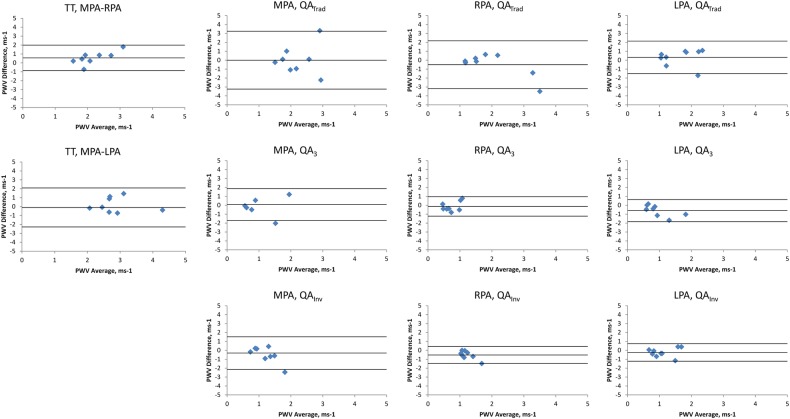
Bland-Altman plots comparing PWV repeatability during the same visit. The middle line represents the mean difference while the upper and lower lines represent the±2SD limit.3, three point technique; Inv, inverse squared reciprocal technique; LPA, left pulmonary artery; MPA, main pulmonary artery; PWV, pulse wave velocity; QA, flow area; RPA, right pulmonary artery; Trad, traditional technique; TT, transit time.

Within-scan reproducibility was assessed in the 20 OHVs. Bland-Altman plots of the between-scan differences are shown in figure 3.

Using the TT technique, the MPA–RPA PWV had better precision but lower accuracy than the MPA–LPA PWV (mean (95% CI) of PWV differences=0.21 (−1.01 to 1.42)/ms and 0.13 (−3.57 to 3.83)/ms for the MPA–RPA and MPA–LPA, respectively). Using the QA technique, the QA_Inv_ technique again consistently yielded improved accuracy and precision over the QA_Trad_ and QA_3_ technique (mean (95% CI) of PWV differences=0.46 (−2.39 to 1.47)/ms, 0.05 (−1.68 to 1.77)/ms and 0.01 (−1.23 to 1.25)/ms for the QA_Trad_, QA_3_ and QA_Inv_ of the MPA, respectively; 0.17 (−0.68 to 1.02)/ms, 0.19 (−1.43 to 1.81)/ms and 0.06 (−0.42 to 0.55)/ms for the QA_Trad_, QA_3_ and QA_Inv_ of the RPA, respectively; and −0.29 (−0.91 to 0.32)/ms, −0.01 (−0.78 to 0.76)/ms and −0.06 (−0.69 to 0.56)/ms for the QA_Trad_, QA_3_ and QA_Inv_ of the LPA, respectively). The high spatial resolution yielded poorer reproducibility than the high temporal resolution sequence (mean (95% CI) of QA_Inv_ PWV differences=0.01 (0.63)/ms and 0.3 (0.86)/ms, respectively). A combination of a high temporal resolution sequence through the RPA combined with the QA_Inv_ post processing yielded the best reproducibility.

### Interscan reproducibility

**Figure 4:**
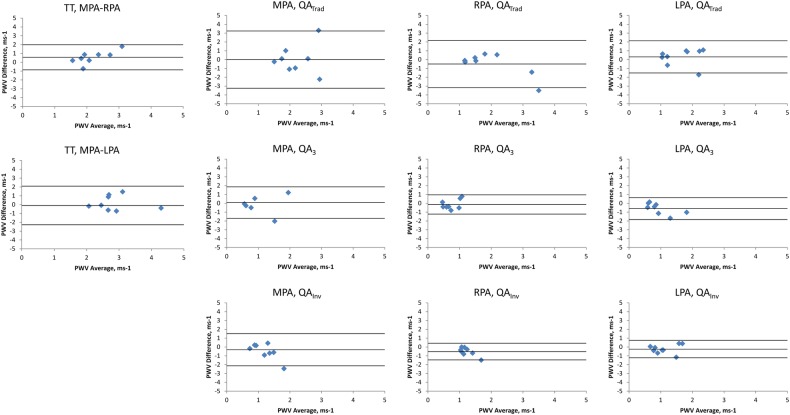
Bland-Altman plots comparing PWV repeatability on separate visits. The middle line represents the mean difference while the upper and lower lines represent the±2SD limit. 3, three point technique; Inv, inverse squared reciprocal technique; LPA, left pulmonary artery; MPA, main pulmonary artery; PWV, pulse wave velocity; QA, flow area; RPA, right pulmonary artery; Trad, traditional technique; TT, transit time.

The scans were repeated at 6 months in 9 of the 10 YHVs. This resulted in reproducible results with no significant differences in the two measurements (p>0.5). Bland-Altman plots of the between-scan differences are shown in figure 4.

Using the TT method, the MPA–RPA PWV had better precision but lower accuracy than the MPA–LPA PWV (mean (95% CI) of PWV differences=0.56 (−0.86 to 1.99)/ms and 0.19 (−1.47 to 1.84)/ms for the MPA–RPA and MPA–LPA, respectively). Using the QA technique, the QA_Inv_ technique again consistently yielded improved accuracy and precision over the QA_Trad_ and QA_3_ technique (mean (95% CI) of PWV differences=0 (−3.24 to 3.24)/ms, −0.07 (−2.11 to 1.96)/ms and −0.43 (−2.18 to 1.32)/ms for the QA_Trad_, QA_3_ and QA_Inv_ of the MPA, respectively; −0.5 (−3.18 to 2.18)/ms, −0.13 (−1.22 to 0.96)/ms and −0.52 (−1.46 to 0.43)/ms for the QA_Trad_, QA_3_ and QA_Inv_ of the RPA, respectively; and 0.31 (−1.51 to 2.14)/ms, −0.59 (−1.84 to 0.67)/ms and −0.24 (−1.22 to 0.75)/ms for the QA_Trad_, QA_3_ and QA_Inv_ of the LPA, respectively). A combination of a high temporal resolution sequence through the RPA combined with the QA_Inv_ post processing yielded the best reproducibility.

### Intraobserver reproducibility

**Figure 5:**
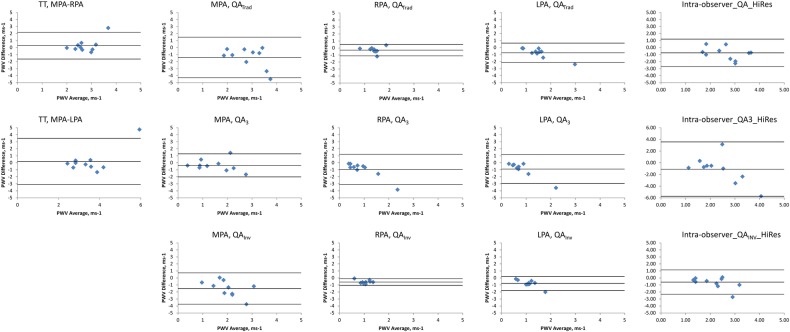
Bland-Altman plots comparing intraobserver PWV repeatability. The middle line represents the mean difference while the upper and lower lines represent the±2SD limit. 3, three point technique; Inv, inverse squared reciprocal technique; LPA, left pulmonary artery; MPA, main pulmonary artery; PWV, pulse wave velocity; QA, flow area; RPA, right pulmonary artery; Trad, traditional technique; TT, transit time.

Bland-Altman plots of the between-scan differences are shown in figure 5. Using the TT method, the MPA–RPA PWV had better precision but lower accuracy than the MPA–LPA PWV (mean (95% CI) of PWV differences=0.28 (−1.63 to 2.19)/ms and 0.19 (−3.11 to 3.48)/ms for the MPA–RPA and MPA–LPA, respectively). Using the QA technique, the QA_Inv_ technique again consistently yielded improved accuracy and precision over the QA_Trad_ and QA_3_ technique (mean (95% CI) of PWV differences=−1.41 (−4.29 to 1.48)/ms, −0.36 (−2.01 to 1.28)/ms and −1.51 (−3.74 to 0.73)/ms for the QA_Trad_, QA_3_ and QA_Inv_ of the MPA, respectively; −0.31 (−1.12 to 0.5)/ms, −0.95 (−3.1 to 1.2)/ms and −0.58 (−1.06 to −0.1)/ms for the QA_Trad_, QA_3_ and QA_Inv_ of the RPA, respectively; and −0.74 (−2.12 to 0.64)/ms, −0.89 (−2.93 to 1.15)/ms and −0.78 (−1.79 to 0.22)/ms for the QA_Trad_, QA_3_ and QA_Inv_ of the LPA, respectively). The high spatial resolution yielded better reproducibility than the high temporal resolution sequence (mean (95% CI) of QA_Inv_ PWV differences=−1.51 (−3.74 to 0.73)/ms and −0.70 (−2.31 to 0.9)/ms for the high temporal resolution and high spatial resolution sequences, respectively). A combination of a high temporal resolution sequence through the RPA combined with the QA_Inv_ post processing yielded the best intraobserver reproducibility.

### Interobserver reproducibility

**Figure 6:**
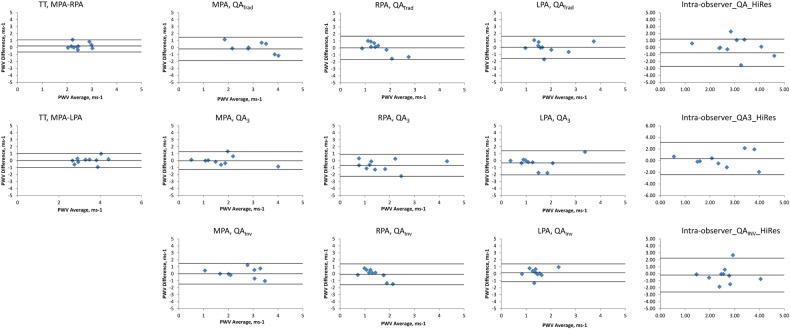
Bland-Altman plots comparing interobserver PWV repeatability. The middle line represents the mean difference while the upper and lower lines represent the±2SD limit. 3, three point technique; Inv, inverse squared reciprocal technique; LPA, left pulmonary artery; MPA, main pulmonary artery; PWV, pulse wave velocity; QA, flow area; RPA, right pulmonary artery; Trad, traditional technique; TT, transit time.

Bland-Altman plots of the between-scan differences are shown in figure 6. Using the TT method, the MPA–RPA PWV had better precision but lower accuracy than the MPA–LPA PWV (mean (95% CI) of PWV differences=0.23 (−0.64 to 1.1)/ms and 0.02 (−0.97 to 1.01)/ms for the MPA–RPA and MPA–LPA, respectively). Using the QA technique, the QA_Inv_ technique again consistently yielded improved accuracy and precision over the QA_Trad_ and QA_3_ technique (mean (95% CI) of PWV differences=−0.2 (−1.86 to 1.47)/ms, 0.02 (−1.25 to 1.29)/ms and 0.01 (−1.47 to 1.5)/ms for the QA_Trad_, QA_3_ and QA_Inv_ of the MPA, respectively; 0.01 (−1.67 to 1.69)/ms, −0.67 (−2.24 to 0.9)/ms and −0.08 (−1.58 to 1.42)/ms for the QA_Trad_, QA_3_ and QA_Inv_ of the RPA respectively; and 0.03 (−1.57 to 1.63)/ms, −0.32 (−2.04 to 1.41)/ms and 0.14 (−1.13 to 1.42)/ms for the QA_Trad_, QA_3_ and QA_Inv_ of the LPA, respectively). The high temporal resolution yielded better reproducibility than the high spatial resolution sequence (mean (95% CI) of QA_Inv_ PWV differences=0.01 (−1.47 to 1.5)/ms and −0.18 (−2.61 to 2.25)/ms for the high temporal resolution and high spatial resolution sequences, respectively). The TT method through the RPA yielded the best overall interobserver reproducibility, while the combination of a high temporal resolution sequence through the RPA combined with the QA_Inv_ post processing yielded the best interobserver reproducibility for the QA technique.

Interobserver and intraobserver variability account for the majority of the interscan variability, with the impact more pronounced in the QA technique than in the TT technique.

### Age

There was no difference between the two groups in terms of age, sex, smoking status, height, weight, BMI or resting heart rate (table 2). However, the older population had a significantly higher resting systolic (YHV 113±1 vs OHV 128±3 mm Hg, p=0.002) and diastolic BP (YHV 68±2 vs OHV 75±2, p=0.028). The pulmonary PWV did not differ between the YHVs and older healthy volunteers using either the TT technique (YHV 2.4±0.3 vs OHV 2.9±0.2, p=0.1) or the QA_Inv_ technique (YHV 0.95±0.1 vs OHV 1.15±0.1, p=0.1). However, a significant difference was observed between the two groups for the aortic arch PWV (YHV 7.4±1.6 vs OHV 10.7±1.6, p=0.014) (table 3).

**Table 2:** Comparison of the demographic and anthropomorphic measures of the two study groups

	YHV	OHV	p Value
Age	31.5±2.4	60.1±1.1	<0.001
Sex (% male)	3 (30)	9 (45)	0.7
Height	1.71±0.02	1.73±0.03	0.8
Weight	76.3±6.5	73.9±3.2	0.7
BMI	25.8±1.9	24.6±0.6	0.5
Current smoker	1 (10%)	3 (15%)	1
Ex-smoker	3 (30%)	6 (30%)	1
Never smoker	6 (60%)	11 (55%)	1
Heart rate	65.3±1.9	64.4±2.8	0.8
Systolic BP	113±1	128±3	0.002
Diastolic BP	68±2	75±2	0.028

BMI, body mass index; BP, blood pressure; OHV, older healthy volunteers; YHV, young healthy volunteers.

**Table 3:** Effects of age on pulmonary and aortic PWV

	YHV	OHV	p Value
Pulmonary PWV			
TT (MPA–RPA)	2.4±0.3	2.9±0.2	0.1
TT (MPA–LPA)	3.0±0.3	3.3±0.3	0.5
MPA QA_Inv_	1.0±0.08	1.2±0.1	0.2
RPA QA_Inv_	1.0±0.06	0.8±0.07	0.1
LPA QA_Inv_	1.0±0.2	0.7±0.05	0.1
Aortic PWV			
Aortic Arch PWV	7.4±1.6	10.7±1.6	0.014

LPA, left pulmonary artery; MPA, main pulmonary artery; OHV, older healthy volunteers; PWV, pulse wave velocity; QA_Inv_, flow area PWV using the inverse squared reciprocal technique; RPA, right pulmonary artery; TT, transit time PWV; YHV, young healthy volunteers.

### Exercise

The isometric calf exercises resulted in a significant and sustained rise in heart rate from 65±2 to 87±3 (p<0.0001) and in BP from 113/68 to 130/84 (p<0.0001). A significant increase in cardiac output was also observed from 5.5±0.4 to 6.7±0.6 L/min (p=0.004) which was mediated through an increase in heart rate rather than an increase in stroke volume (p=0.98). Exercise resulted in erroneous measures in two participants using the TT technique and in one using the QA technique.

Using the TT technique, the MPA–RPA PWV changed from 2.43±0.26 at rest to 2.86±0.25 during exercise (mean (95% CI) of PWV differences=−0.42 (−1.2 to 0.4)/ms, p=0.24); while the MPA–LPA PWV changed from 3.03±0.27 at rest to 3.35±0.39 during exercise (mean (SD) of PWV differences=−0.32 (−1.6 to 1)/ms, p=0.57).

Using the QA_Inv_ technique: the MPA PWV went from 0.95±0.09 at rest to 0.91±0.1 during exercise (mean (95% CI) of PWV differences=0.03 (−0.2 to 0.3)/ms, p=0.77); the RPA PWV went from 1.03±0.06 at rest to 0.92±0.17 during exercise (mean (95% CI) of PWV differences=0.11 (−0.3 to 0.5)/ms, p=0.53); the LPA PWV went from 1.03±0.16 at rest to 1.05±0.11 during exercise (mean (95% CI) of PWV differences=−0.02 (−0.4 to 0.3)/ms, p=0.9).

## Discussion

We have shown that (1) the QA method using a high temporal resolution sequence through the RPA using a calculation that accounts for wave reflections yields consistently better within-scan, interscan, intraobserver and interobserver reproducibility; (2) age-related arterial stiffening as seen in the systemic circulation does not occur in the pulmonary vessels; (3) pulmonary PWV is stable and consistent at rest and exercise.

This is the most comprehensive analysis of the techniques for measuring pulmonary PWV to date. Prior studies have either provided within-scan reproducibility in n=10 using just the TT technique,^25^ within and between-scan reproducibility in n=17 using just the QA technique^26^ or interobserver comparison but without interscan assessment in n=33.^24^ Thus our study of measurement techniques as well as interrogating the effects of different acquisition sequences and different postprocessing methods provides the first in-depth and rigorous assessment of pulmonary PWV assessment.

It is perhaps not surprising that the RPA QA PWV measurements were the most precise as the RPA suffers from significantly less through plane motion during the cardiac cycle than the MPA as well as suffering from less respiratory variation in location. However, justification of focusing on one of the branch PAs, rather than the MPA, relies on changes affecting all three arteries equally. While it may be reasonable to assume this in diffuse pulmonary disease states such as chronic obstructive pulmonary disease (COPD) or idiopathic pulmonary arterial hypertension, prior work in chronic thromboembolic pulmonary hypertension (CTEPH) has demonstrated that the capacitance varies between the LPA and RPAs.^30^ Thus, while the RPA may provide the best results in terms of reproducibility, care may have to be taken when using this in disease states, particularly CTEPH where despite its greater intermeasure variability the MPA may be better suited. Further work is required in other disease states to evaluate the degree of variability in measures of pulmonary stiffness. Further consideration must be given to the fact that there appears to be a change in stiffness between the main pulmonary trunk and the branch PAs observed in our study with the RPA and LPAs exhibiting lower stiffness than the MPA. Current studies demonstrating prognostic significance of proximal pulmonary arterial stiffness have all focussed on changes in the MPA; thus, these findings will have to be replicated in the RPA in order to validate its usefulness as a marker of arterial stiffness of prognostic significance.

Our finding of improved reproducibility with a lower spatial resolution and higher temporal resolution is slightly counter intuitive as it has previously been thought that high spatial resolution was the more important factor in QA assessment due to the need for accurate area measurement.^24^ ^27^ However, it may be that increasing the temporal resolution increases the data points for deriving the flow area gradient, thus improving the reproducibility. Indeed the QA_Inv_, which uses all the points in early systole, was seen to be more precise than the QA_3_ technique which uses only the first three. The good agreement of QA_Inv_ and QA_3_ compared with QA_Trad_, despite their different sampling windows and different calculations, also shows the importance of correcting for the reflected wave in early systole as they both produced significantly lower results than the QA_Trad_ using the high spatial resolution and high temporal resolution sequences. Previous animal models have shown an early expansion wave arriving during systole in the MPA,^31^ ^32^ and this becomes even more important in disease when a backward compression wave starts to arrive in systole.^33^ Recently, a phase contrast acquisition technique using a golden angle radial acquisition has been described in the pulmonary circulation which maintains high edge sharpness while maintaining good temporal resolution which may further improve the reproducibility of this technique.^28^ Given that segmentation errors affect the area and flow measurements, it would be assumed that this would result in lower reproducibility than the TT technique, especially as previous studies in the aorta have shown exactly this.^24^ ^27^ It is likely that due to the short path length inherent in measuring PWV in the pulmonary circulation, that small changes in RR variability and flow states result in greater impact on the short TTs compared with the relatively longer TTs in the aorta where imaging planes can be positioned at significantly greater distances apart.

In our study, we found no change in pulmonary arterial PWV with exercise. This is in contradistinction to the recent findings of Forouzan *et al*^34^ who demonstrated an increase in PWV on exercise in a study of n=15 using the QA technique. This previous study obtained a larger increase in cardiac output compared with our own and was larger than our current exercise group (n=10); thus, it may be that in our study the participants were insufficiently stressed to illicit a change in the proximal pulmonary arterial stiffness or that the sample size was too small. However, there are several reasons to support the accuracy of the findings of the current study. First, our measures were obtained during rather than after exercise, and we have used the TT and QA technique with a similar lack of change seen with both. Second, the previous study used the QA_Trad_ method for calculating the data, which we have shown provides a higher measurement of PWV than methods accounting for wave reflections. Wave reflections are known to increase substantially during exercise states, thus the change in their PWV may have been due to a change in the magnitude of wave reflections rather than a change in arterial stiffness.^32^ Finally, our observations are in agreement with two prior invasive studies: the first of these by Laskey *et al*^35^ using invasive pressure and velocity wires to calculate the arterial input impedance spectrum demonstrated no change in PWV with exercise in healthy controls; and a second by Domingo *et al*^36^ using right heart catheterisation and intravascular ultrasound demonstrating an increased pulsatility but with a fall in elastic modulus and no significant change in capacitance on exercise.

We have shown a lack of change in the pulmonary arterial PWV with age in comparison with the aorta where the expected increase in PWV was observed. This is in contradistinction to a recent study by Dawes *et al*^37^ which demonstrated a correlation between pulmonary PWV and age in a cohort of n=156. The fact that using the same technique in the systemic circulation we were able to observe a significant change in aortic PWV suggests our technique is robust, but that the changes with age in the pulmonary circulation are far smaller than those within the aorta, and thus, require far larger populations to detect.

Several limitations must be mentioned regarding this study. First, no gold standard was used to compare the techniques against. The main reason for this is that the gold standard is invasive right heart catheterisation, which in a healthy population with no comorbidities would be difficult to justify. However, use of the technique in patients due to undergo a clinically indicated right heart catheterisation would be a useful avenue for future work. In addition, while none were compared with the gold standard, reproducibility and reduced interscan variability is as, if not more, important than accuracy as it allows detection of smaller changes within the study group or patient cohort of interest. Second, a high spatial resolution sequence was not applied through either the RPA or LPA, thus while it is unlikely given the trend of results seen in the MPA, it cannot be excluded that higher spatial resolution sequences through these regions would not result in improved reproducibility compared with the high temporal resolution sequences. Third, while the number undergoing repeat measurement while on table was reasonably sized, those undergoing repeat measurement at 6 months were substantially smaller; however, the results of this largely mirrored the results of the on-table repeat measure, thus aiding in the validation of their findings.

In conclusion, use of the QA technique through the RPA combined with a high temporal resolution acquisition and a postprocessing technique to account for wave reflections yields the most reproducible measurements of pulmonary PWV.
